# Risk factors for one-year mortality following discharge in patients with acute aortic dissection: development and validation of a predictive model in a cross-sectional study

**DOI:** 10.1186/s12872-024-03766-6

**Published:** 2024-02-29

**Authors:** Ting Zhou, Jing-Xiao Li, Chao-Yong Zhang, Yu-Gui Li, Jun Peng, Chun-Lou Wei, Meng-Hua Chen, Hua-Fu Zhou

**Affiliations:** 1 Cardiothoracic Surgery Intensive Care Unit, The First Affiliated Hospital of Guangxi Medical University, Nanning, Guangxi P.R. China; 2 Department of Thoracic Surgery, The First Affiliated Hospital of Guangxi Medical University, Nanning, Guangxi P.R. China; 3 The First Affiliated Hospital of Guangxi Medical University Coronary Care Unit, Nanning, Guangxi P.R. China; 4 Department of Cardiac Surgery, The First Affiliated Hospital of Guangxi Medical University, Nanning, Guangxi P.R. China; 5 The Second Affiliated Hospital of Guangxi Medical University Intensive Care Unit, Nanning, Guangxi P.R. China

**Keywords:** Acute aortic dissection, Public database, Risk prediction model

## Abstract

**Purpose:**

This study was aimed to identify the risk factors that influence the mortality risk in patients with acute aortic dissection (AAD) within one year after discharge, and aimed to construct a predictive model for assessing mortality risk.

**Methods:**

The study involved 320 adult patients obtained from the Medical Information Mart for Intensive Care (MIMIC) database. Logistic regression analysis was conducted to identify potential risk factors associated with mortality in AAD patients within one year after discharge and to develop a predictive model. The performance of the predictive model was assessed using the receiver operating characteristic curve (ROC), calibration curve, and decision curve analysis (DCA). To further validate the findings, patient data from the First Affiliated Hospital of Guangxi Medical University (157 patients) were analyzed.

**Results:**

Univariate and multivariate logistic regression analyses revealed that gender, length of hospital stay, highest blood urea nitrogen (BUN_max), use of adrenaline, and use of amiodarone were significant risk factors for mortality within one year after discharge (*p* < 0.05). The constructed model exhibited a consistency index (C-index) and an area under the ROC curve of 0.738. The calibration curve and DCA demonstrated that these indicators had a good degree of agreement and utility. The external validation results of the model also indicated good predictability (AUC = 0.700, *p* < 0.05).

**Conclusion:**

The personalized scoring prediction model constructed by gender, length of hospital stays, BUN_max levels, as well as the use of adrenaline and amiodarone, can effectively identify AAD patients with high mortality risk within one year after discharge.

## Central message

In this article, the personalized scoring prediction model constructed by gender, length of hospital stays, highest blood urea nitrogen, as well as the use of adrenaline and amiodarone, can effectively identify acute aortic dissection patients with high mortality risk within one year after discharge.

## Introduction

The incidence rate of acute aortic dissection (AAD) in cardiovascular diseases is relatively low at 0.0047%. Despite of that, AAD poses a significant threat to patients, as its annual mortality rate exceeds 20% [1]. As established by Stanford University in 1970, AAD is diagnosed into two primary types: type A (ascending aortic disease) and type B (descending or abdominal aortic disease) [2]. Currently, medical treatment, interventional therapy, and surgical treatment are the main approaches employed to address AAD [3]. However, the all-cause mortality rate for AAD patients can still reach 15% [4]. D-dimer serves as a highly sensitive indicator for the detection of AAD and plays a crucial role in evaluating treatment efficacy in clinical practice [5]. However, relying solely on a single indicator may not provide a reliable criterion for assessing treatment effectiveness or predicting patient survival time. To address this limitation and evaluate the severity of a patient’s condition as early as possible, researchers have proposed various in-hospital and out-of-hospital evaluation scales. The Aortic Dissection Risk Score (ADD-RS) is a highly sensitive tool that determines the presence of AAD by taking into account factors such as clinical manifestations, blood pressure, family history, and medical history. It facilitates the identification of AAD occurrence prior to initial diagnosis and treatment [6]. Tashima et al. utilized the Agaston score to assess postoperative AAD patients and discovered that those with a high grouping score faced a greater risk of thrombosis, potentially leading to adverse outcomes such as cardiac and pulmonary embolism [7].

Furthermore, some researchers have developed models aiming to predict the risk of death for hospitalized patients. These models primarily involved indicators such as age, Marfan syndrome, treatment methods, blood pressure, and complications, among others [8]- [9]. Notably, existing risk scoring models and assessment scales primarily focus on predicting the risk of in-hospital mortality, with no literature reports available on the risk of patient outcomes after discharge. This lack of information hampers the evaluation of treatment effectiveness and follow-up interventions after discharge. The risk of death after discharge, particularly within one year, remains a concern in clinical practice. Therefore, it is necessary to investigate the risk factors influencing the death of AAD patients within one year after discharge and establish a personalized scoring-based predictive model.

In this study, we conducted an analysis of 320 AAD samples from public databases and 157 AAD samples from the First Affiliated Hospital of Guangxi Medical University. Our objective was to explore the risk factors affecting the death of AAD patients within one year after discharge. Additionally, we constructed a predictive model to assess the risk of death for AAD patients within one year after discharge.

## Methods

### Ethical statement

The present study had been approved by Ethics Review Committee of the First Affiliated Hospital of Guangxi Medical University (approval number: 2023-E489-01). All patients were aware of the purpose and method of this study and have signed an informed consent form for sample use.

### Sample sources for prediction models

This multicenter retrospective study analyzed adult patients from the Medical Information Mart for Intensive Care (MIMIC) database. The objective was to construct a predictive model for assessing the risk of death among patients with AAD within one year after discharge. The project received was approved by the Institutional Review Committee of the Massachusetts Institute of Technology and Beth Israel Deaconess Medical Center (certificate number 46,205,578). Informed consent was waived. The MIMIC database, developed by the Massachusetts Institute of Technology, contains demographic information, laboratory test results, and intravenous drug records. The database has been updated to the MIMIC-iv version, and the sample collection period is from 2008 to 2019 [10]. The inclusion criteria for the samples were as follows: (1) Patients over the age of 18; (2) Patients diagnosed with “acute aortic dissection (AAD)” upon admission; (3) Patients with first-time admission due to AAD. The exclusion criteria were as follows: (1) Patients under the age of 18; (2) Patients previously treated for AAD.

### Primary outcome

The objective of this study was to develop a predictive model for assessing the risk of death among patients within one year after discharge. Death information for discharged patients was obtained from the US Social Security Death Index. The duration between the date of death and date of discharge was calculated for these patients. Patients with a duration of less than 365 days were included in the death group, while the remaining patients, including non-deceased patients, were included in the control group.

### Collection and processing of clinical information

This study included a sample of 320 patients with AAD. Demographic information was recorded, including age (mean ± standard deviation [SD]: 69.03 ± 14.47 years), height (mean ± SD: 168.14 ± 12.08 cm), weight (mean ± SD: 79.07 ± 20.90 kg), and BMI (mean ± SD: 27.86 ± 6.35). A detailed summary is presented in Table 1. Additionally, patients’ data was collected throughout the study period, including gender, admission and discharge times, race, time of death (if applicable), medical history of hypertension, coronary heart disease, diabetes, and Marfan syndrome, as well as ICU admission. Further measurements included the highest systolic blood pressure (SBP) after admission, red blood cell (RBC) count, hemoglobin count, white blood cell (WBC) count, platelet (PLT) count, blood creatinine, blood urea nitrogen (BUN), as well as blood concentrations of potassium, calcium, and magnesium. The use of adrenaline, dobutamine, and amiodarone was also recorded. All data collected constituted the variables analyzed in this study. Outliers (such as samples with many missing values, as well as some misclassified values, etc.) were removed, and missing values were supplemented from the collected data. Independent sample t-tests were used to analyze the continuous variable data in this dataset, while χ^2^ tests were used to analyze binary variable data.

**Table 1:** Demographic characteristics of the samples included from the MIMIC database

Variable	Mean	SD
Age (years)	69.025	14.47014
Height (cm)	168.1419	12.07874
Weight (kg)	79.0733	20.90327
BMI	27.8568	6.34865

SD: Standard deviation; BMI: Body mass index

Subsequently, continuous variable data was transformed into binary variables for further analysis. Patients aged 60 years or older at the time of treatment were recorded as “elderly”. Patients with a BMI > 23.9 were recorded as “overweight”. Due to the variable treatment duration for AD patients, the median of the hospitalization length (137 h) was adopted as the dividing line between “prolonged hospital stay” and “short hospital stay”. Patients who self-identified as white were recorded as “white race”, and otherswere all categorized into “other races”. According to whether the max SBP (BP_max) greater than or equal to 140 mmHg, patients after admission are divided into hypertension group and non-hypertension group. According to whether the minimum red blood cell count (RBC_min) < 3.5 × 1012/L or a minimum hemoglobin value (Hb_min) < 110 g/L, patients are divided into anemic group and non-anemic group. According to whether the minimum platelet count (PLT_min) < 100 × 109/L, patients are divided into low-platelet group and non-low-platelet group. According to whether the highest value of creatinine in their blood (Scr_max) greater than 133 µmol/L or the highest value of blood urea nitrogen (BUN_max) greater than 20 mg/dL, patients are divided into reduced-renal-function group and non-reduced-renal-function group. According to whether the lowest blood potassium concentration (K_min) < 3.5 mmol/L, patients are divided into hypokalemia group and non-hypokalemia group. According to whether the highest blood potassium concentration (K_max) > 5.5 mmol/L, patients are divided into hyperkalemia group and non-hyperkalemia group. According to whether the lowest blood calcium concentration (Ca_min) < 8.5 mg/dL, patients are divided into hypocalcemia group and non-hypocalcemia group. According to whether the highest blood calcium concentration (Ca_max) > 10.5 mg/dL, patients are divided into hypercalcemia group and non-hypercalcemia group. According to whether the lowest blood magnesium concentration (Mg_min) < 2.64 mg/dL, patients are divided into hypomagnesemia group and non-hypomagnesemia group. According to whether the highest blood magnesium concentration (Mg_max) > 5.04 mg/dL, patients are divided into hypermagnesemia group and non-hypermagnesemia group. The new binary variable dataset obtained through the above transformation will be used for subsequent logistic regression analysis.

### Screening of risk factors and construction of nomogram

Before analysis, we randomly selected 70% of the samples from the transformed binary variable dataset for logistic regression analysis. The objective was to examine the relationship between the variables and the survival status of patients within one year after discharge. The remaining 30% of the samples were reserved for internal cross-validation, where the nomogram’s performance was evaluated on independent data. The odds ratio (OR), 95% confidence interval (95% CI), and *p*-value of each factor were obtained. Significant factors (*p*-value < 0.05 and 95% CI of OR not including 1) were considered potential mortality risk factors for AAD patients within one year after discharge. Multivariate logistic regression analysis was then used to establish a risk prediction model. In the risk prediction model, each risk factor was assigned a certain weight, which directly affected the evaluation score. The percentage corresponding to the total score ultimately represented the mortality risk within one year after discharge.

### Evaluation of risk prediction models

#### Internal validation

To assess the validity and dependability of the model, the consistency index (C-index) was calculated. Besides, a receiver operating characteristic curve (ROC) was plotted to evaluate the model’s predictive capacity, with a higher area under the curve (AUC) indicating greater predictive ability, consistent with the C-index. In addition, a calibration curve was generated to assess the model’s goodness of fit by comparing predicted probabilities to actual probabilities. Lastly, decision curve analysis (DCA) was utilized to assess the practicality of various risk factors.

#### External validation

External validation of the identified risk factors was conducted by collecting data from patients with AD who visited the First Affiliated Hospital of Guangxi Medical University between January 1, 2021, and December 31, 2021. Through calling patients and their families, we aim to determine the patient’s survival status within one year after discharge, as well as the accurate timing of clinical outcomes (death), to ensure the accuracy of our data and outcome records [11]. Prior to further analysis, both BUN_Max and length of hospitalization were considered continuous variables. BUN_Max was processed using the aforementioned method. The length of hospitalization was categorized by the median length of hospital stay (312 h) of the patient group. Patients with a hospital stay of ≤ 312 h were classified as the “short-stay population,” whereas those with a hospital stay > 312 h were classified as the “long-stay population.” To assess the generalizability of the previously established nomogram, the C-index was calculated, and the ROC was plotted.

### Statistical analysis

Estimate the sample size using GPower 3.9.1.7. When α = 0.05, 1- β = When 0.95 and effect size = 0.5, the sample sizes of the two groups should be at least 54 and 218, and the total sample size should be at least 80. All source codes for the assessment of MIMIC database data in this study could be found at the website: https://gitcode.net/mirrors/MIT-LCP/mimic-iv. Independent sample t-tests and χ^2 tests were conducted using IBM SPSS 23.0 to calculate mean, SD, and *p*-value. The rms package, foreign package, and rmda package of R 3.6.2 were used to draw the nomogram, calibration curves, ROC curves, and DCA curves. In this study, a *p*-value < 0.05 was considered statistically significant.

## Results

### Characteristics of patients and disease

**Fig. 1:**
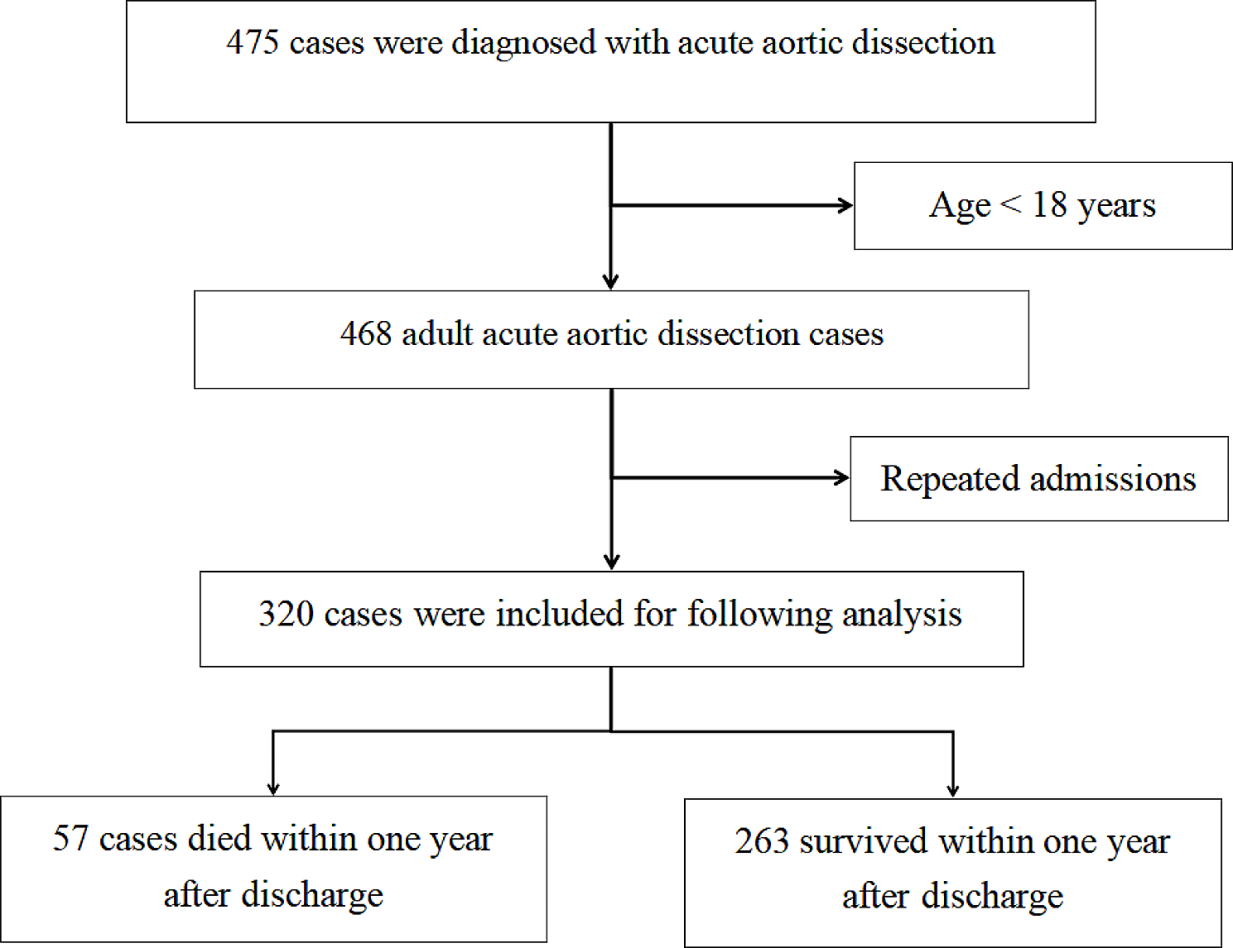
Study enrollment flowchart

A total of 475 cases were diagnosed with AAD (Table 1). After excluding minors (age < 18 years) and repeated admissions, 320 patients were selected for inclusion in this study (Fig. 1). After calculating using GPower 3.1.9.7, our sample size has good reliability, and a sufficient sample size eliminates the possible sparse effect in subsequent regression analysis [12]. Notably, significant differences (*p* < 0.05) were observed between the death and control groups regarding age, weight, BMI, RBC_Min, Hb_Min, WBC_Min, Ca_Min, as well as the administration of adrenaline and amiodarone. There was no significant statistical difference in height, Length of hospital stays, BP_max, WBC_max, PLT_max, PLT_min, Scr_max, BUN_max, K_max, K_min, Ca_max, Mg_max, Mg_min, gender, race, hypertension, coronary arteriosclerosis, diabete, transferd to ICU and dobutamine using between the two groups of patients (Tables 2 and 3).

**Table 2:** Comparison of econometric data between the death group and the control group from the MIMIC database

Clinical data	Death group (*n* = 57)		Control group (*n* = 263)		t	*p*-value
	Mean	SD	Mean	SD		
Age (years)	74.720	14.675	67.790	14.154	-3.329	0.001
Height (cm)	167.180	12.320	168.350	12.039	0.665	0.507
Weight (kg)	73.910	20.234	80.190	20.915	2.067	0.039
BMI	26.330	6.370	28.190	6.307	2.009	0.045
Length of hospital stays (hours)	175.630	177.615	217.430	211.685	1.388	0.166
BP_max	154.920	7.329	156.320	18.443	0.936	0.350
RBC_min	2.975	0.818	3.225	0.798	2.132	0.034
Hb_min	8.660	2.719	9.520	2.636	2.205	0.028
WBC_max	16.230	10.323	13.391	6.970	-1.981	0.052
WBC_min	8.167	3.729	7.073	2.452	-2.117	0.038
PLT_max	295.190	162.784	319.670	155.340	1.069	0.286
PLT_min	152.390	106.418	161.310	81.691	0.596	0.553
Scr_max	2.220	1.644	1.950	2.290	-0.858	0.391
BUN_max	89.220	185.631	83.100	185.241	-0.226	0.821
K_max	5.280	1.192	6.940	32.887	0.381	0.703
K_min	3.540	0.647	3.470	0.468	-0.779	0.439
Ca_max	9.580	3.399	9.150	1.111	-0.95	0.346
Ca_min	7.970	0.907	8.220	0.731	2.004	0.049
Mg_max	2.540	0.540	2.440	0.452	-1.425	0.155
Mg_min	1.910	0.319	1.860	0.246	-1.128	0.263

SD: Standard deviation; BMI: Body mass index; BP_ Max: The highest blood pressure during hospitalization; RBC_ Min: minimum value of red blood cell count; Hb_ Min: The lowest value of hemoglobin; WBC_ Max: the highest value of white blood cells; WBC_ Min: the lowest value of white blood cells; PLT_ Max: the highest value of platelets; PLT_ Min: minimum platelet count; Scr_ Max: the highest value of blood creatinine; BUN_ Max: the highest value of blood urea nitrogen; K_ Max: the highest value of potassium ions; K_ Min: minimum value of potassium ions; Ca_ Max: the highest value of calcium ions; Ca_ Min: minimum value of calcium ions; Mg_ Max: the highest value of magnesium ions; Mg_ Min: The lowest value of magnesium ions

**Table 3:** Comparison of count data between death groups and control groups from the MIMIC database

Clinical data	Samples of death group	Samples of control group	*p*-value
**Gender**			
Male	30	164	0.173
Female	27	99	
**Race**			
White	40	173	0.524
Other	17	90	
**Hypertension**			
Yes	32	164	0.382
No	25	99	
**Coronary arteriosclerosis**			
Yes	14	43	0.142
No	43	220	
**Diabete**			
Yes	7	35	0.835
No	50	228	
**Marfan syndrome**			
Yes	0	3	0.418
No	57	260	
**Retransfered to ICU**			
Yes	34	157	0.995
No	23	106	
**Used Adrenaline**			
Yes	17	46	0.034
No	40	217	
**Used Dobutamine**			
Yes	2	8	1.000
No	55	255	
**Used Amiodarone**			
Yes	10	80	0.049
No	47	183	

ICU: Intensive Care Unit

### Variable screening and nomogram construction

**Fig. 2:**
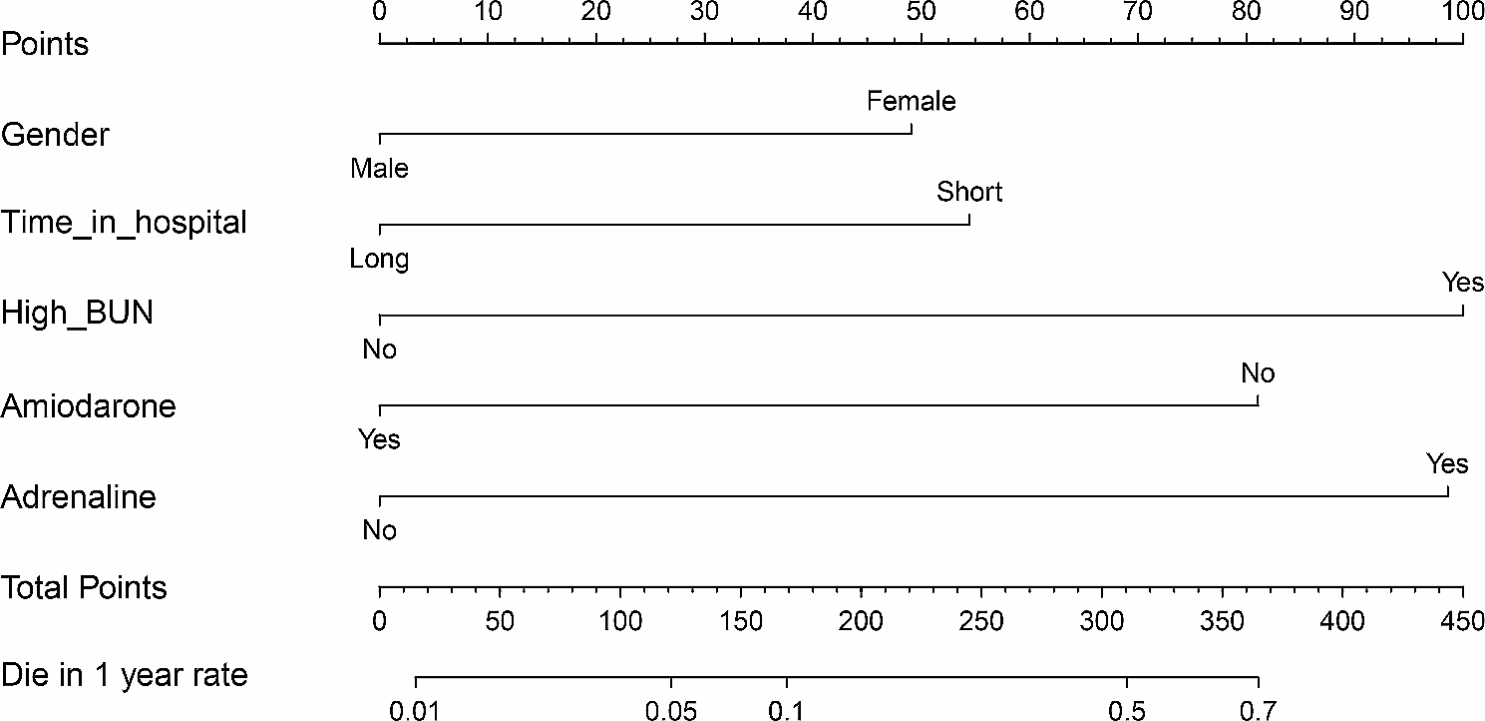
Nomogram constructed based on potential risk factors

**Fig. 3:**
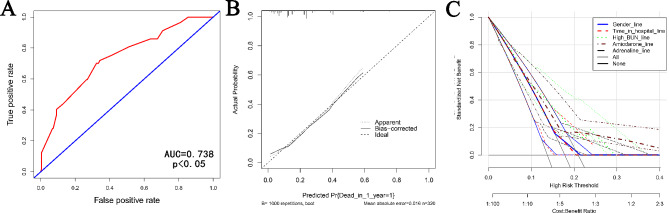
ROC (**A**), calibration curve (**B**), and DCA curve (**C**) for internal validation of nomogram

Univariate logistic regression analysis revealed that gender, length of hospital stay, BUN_Max, use of adrenaline, and use of amiodarone were significant risk factors for mortality within one year after discharge (*p* < 0.05). Subsequent multivariate regression analysis confirmed the strong predictive value of these factors for mortality within one year after discharge (*p* < 0.05), as shown in Table 4. Based on these findings, we constructed a nomogram using a nomogram to score each risk factor. Specifically, male gender was scored as 0 points, while female gender was scored as 48 points. Long hospitalization time (> 137 h) was scored as 0 points, while short hospitalization time (≤ 137 h) was scored as 58 points. Low BUN_Max (< 20 mg/dL) was scored as 0 points, while high BUN_Max (≥ 20 mg/dL) was scored as 100 points. Use of amiodarone during hospitalization was scored as 0 points, while non-use was scored as 81 points. Use of adrenaline during hospitalization was scored as 96 points, while non-use was scored as 0 points. The total score was calculated as the sum of these scores, corresponding to the probability of death within one year (170 points corresponded to a 10% probability, 320 points corresponded to a 50% probability, and 365 points corresponded to a 70% probability), (Fig. 2). Through internal cross validation, the AUC for nomogram was 0.738, indicating its strong predictive ability for the risk of death in AAD patients within one year after discharge (Fig. 3A).

**Table 4:** Potential risk factors for death within one year after discharge in AAD patients screened through univariate and multivariate logistic analysis

Term	Univariate analysis		Multivariate analysis	
	Odds Ratio (95% CI)	*p*-value	Odds Ratio (95% CI)	*p*-value
Gender	0.466 (0.247–0.882)	0.019	0.450 (0.214–0.931)	0.033
Length of hospitalization	0.429 (0.213–0.862)	0.017	0.256 (0.093–0.672)	0.007
BUN_max	4.736 (2.011–11.154)	< 0.001	3.477 (1.423–9.366)	0.009
Amiodarone	0.283 (0.119–0.677)	0.005	0.220 (0.077–0.560)	0.003
Adrenaline	4.632 (2.083–10.300)	< 0.001	3.640 (1.443–9.423)	0.007

95% CI: 95% confidence interval; BUN_ Max: The highest value of blood urea nitrogen

### Validation of nomogram

To further assess the nomogram’s performance, a calibration curve was generated, demonstrating a strong agreement between the nomogram’s predicted values and the actual observed values (Fig. 3B). Additionally, we employed the DCA curve to evaluate the practical utility of the various indicators. As shown in Fig. 3C, when the colored line segment is located within the triangle constructed by the gray and black lines, as well as the Y-axis, it is reasonable to believe that the corresponding item of the line segment has the potential to predict the mortality risk of AAD patients one year after discharge. The curves corresponding to all indicators incorporated into the nomogram exceeded the extreme curves (gray and black), indicating that these indicators possess a great degree of utility.

**Fig. 4:**
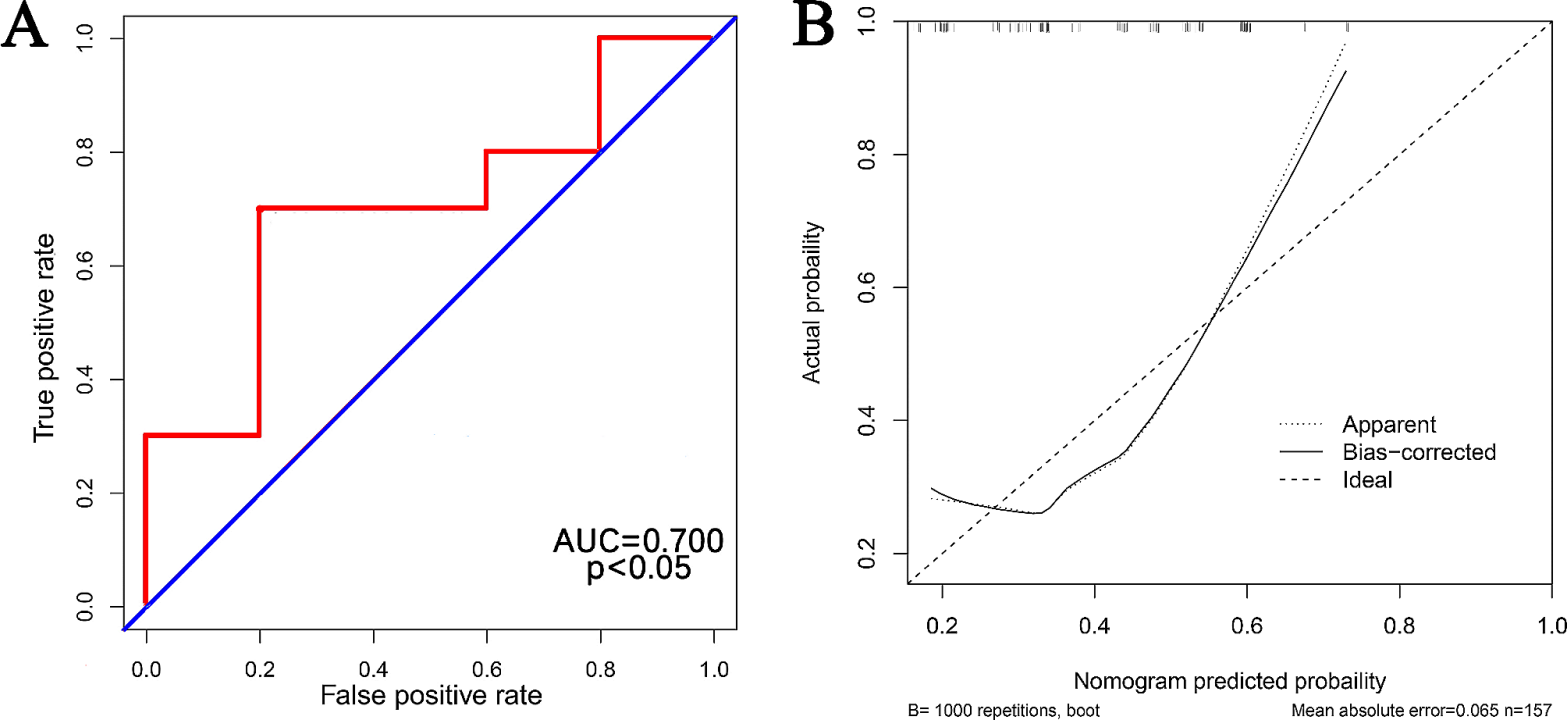
ROC and calibration curve for external validation

Furthermore, we incorporated data from 157 patients with AD from the First Affiliated Hospital of Guangxi Medical University (Table 5), and calculated the area under the C-index and ROC curves to be 0.700, suggesting that the developed nomogram in this study exhibited good predictive performance, it also has good applicability to patients from other centers (Fig. 4A and B).

**Table 5:** Overview of data from the First Affiliated Hospital of Guangxi Medical University

Term	Samples of death group	Samples of control group
**Gender**		
Male	39	70
Female	21	27
**Time_in_hospital**		
Long	33	34
Short	27	63
**BUN_max**		
Normal	9	22
Abnormal	51	75
**Adrenaline**		
Yes	48	80
No	12	17
**Amiodarone**		
Yes	41	14
No	56	46

BUN_ Max: The highest value of blood urea nitrogen

## Discussion

AAD is a severe condition affecting the aorta. Although the incidence rate of AAD is relatively low, patients diagnosed with this condition generally have a poor prognosis [13]. The high mortality rate associated with AAD often leads clinical practitioners to prioritize managing patients’ conditions during their hospital stay, potentially neglecting their long-term prognosis after discharge. With advancements in technology, we have the capacity to intervene in the early stages of AAD and slow down its progression at present. However, in China, there is a lack of post-discharge follow-up on patients, leading to neglect of their rehabilitation and subsequent treatment, which can worsen the disease after initial treatment [14]. Therefore, it is crucial to assess the risk of postoperative outcomes for AAD patients based on their hospitalization and establish a comprehensive follow-up system. It could enhance the recovery rate of discharged patients and reduce the medical burden on them and their families. Thus, we identified five potential risk factors and developed a risk prediction model for one-year post-discharge mortality, facilitating early identification of patients at risk.

Traditionally, researchers have observed gender disparities in the incidence of AAD. Males have a higher prevalence of AAD compared to females and tend to present with a wider range of aortic lesions [15, 16]. Additionally, male patients are more likely to be diagnosed with complications such as inadequate blood supply to vital organs [17]. Conversely, although the condition was reported to be less severe in females, they may have a lower attributable mortality rate for AD and its complications, consistent with our findings [18]. In our risk prediction model, both univariate and multivariate logistic regression analyses revealed that gender strongly predicts the mortality risk within one year. Female patients were assigned a higher score in our risk prediction model, indicating the need for healthcare professionals to prioritize monitoring their disease progression after discharge and intervene as necessary. To understand the elevated mortality rate of female patients after discharge, we conducted a literature search. In the clinical diagnosis and treatment of heart disease, patients of different genders also have different tendencies towards treatment methods. Female patients are more inclined to choose transcatheter aortic valve implantation for the treatment of valvular heart disease, while the willingness to choose interventional surgery for ischemic heart disease is significantly lower than that of males [19]. Different options bring different outcomes. Although there is currently no clear research indicating significant gender differences in the selection of treatment options and clinical prognosis for AAD, some studies have provided us with clues. Existing studies showed that male patients experience more frequent chest pain symptoms in the early stages of AAD, while female patients often present with poor peripheral vascular perfusion and neurological dysfunction before receiving treatment. The presence of invisible early symptoms and severe complications contributes to worse conditions at the time of treatment, worsening their prognosis [20]. Moreover, female patients often have smaller arterial diameters, making them more susceptible to the impact of AAD on fine blood vessels, further deteriorating their prognosis [21]. Therefore, it is crucial to focus not only on the in-hospital development of AAD patients but also on improving the survival rates of female patients after discharge.

In our research, we found that the duration of hospitalization could be a potential risk factor for one-year post-discharge mortality. Longer hospital stays were associated with a decreased risk of death. The choice of therapeutic approaches for different types of AAD varies, with surgery being the optimal option to extend patients’ lives and improve their prognosis [3]. Surgery leads to superior treatment outcomes compared to internal medicine treatment, but it also requires longer hospital stays. Patients opting for internal medicine treatment need extended hospital stays to assess drug efficacy and mitigate side effects. Furthermore, AAD often leads to early-onset complications, and an extended hospitalization period allows clinical physicians to manage these complications in a timely and effective manner, which significantly improves patient prognosis [22].

BUN is a crucial indicator reflecting renal function and plays a vital role in the occurrence and progression of cardiovascular diseases such as heart failure [23]. Liu’s research demonstrated that BUN is an independent mortality predictor during hospitalization among AAD patients, with higher BUN levels corresponding to a higher risk of hospital mortality, consistent with our findings [24]. In the predictive model we constructed, a BUN level > 20 mg/dL during hospitalization was assigned a score of 100, indicating that BUN is the most influential factor in nomogram for the risk of death within one year after discharge. Inadequate visceral perfusion is one of the most severe complications following the onset of AAD, and it has a significant impact on renal function [25]. Ischemia-induced kidney damage may impair the elimination of BUN from the body, resulting in increased BUN levels. Therefore, early detection and prediction of potential renal function damage, along with early intervention, are advantageous for improving the prognosis of AAD patients [24]. Additionally, our predictive model showed that the use of adrenaline somewhat increased the risk of death within one year after discharge. Although adrenaline plays a crucial role in overcoming hypotension during the onset of AAD, its side effects deserve attention. Adam et al. reported a case of an electrical storm induced by the use of adrenaline after AAD surgery, as it can cause or exacerbate arrhythmias [26]. Wang et al.‘s research also demonstrated that high concentrations of catecholamines can promote the expansion of the false lumen, thereby increasing the risk of rupture [27]. Furthermore, adrenaline directly stimulates glomerular contraction, leading to reduced renal blood supply in AAD patients, ultimately increasing BUN levels [28]. Hence, comprehensive assessment of visceral perfusion and function before administering vasopressors, particularly adrenaline, is essential.

Arrhythmias are the most common cardiac rhythm changes observed after AAD and are also a frequent cause of hemodynamic disorders associated with AAD [29]. Currently, there has been no literature directly indicating the relationship between arrhythmia and short-term mortality of AAD patients. However, in nomogram, the use of amiodarone was found to be beneficial for improving the one-year survival rate of patients after discharge. Unfortunately, due to unavailability of medical records from the MIMIC database, we couldn’t determine the specific indications for administering amiodarone to these patients. Further research is required to explore the relationship between amiodarone and short-term mortality in AAD patients.

Our results indicated that gender, length of hospital stay, BUN_Max, use of adrenaline, and use of amiodarone were potential risk factors for death within one year after discharge in AAD patients. These results emphasize some important factors that need to be noted in the management of AAD patients. For patients of different genders, clinical doctors need to assess the progression of the disease in more detail. Meanwhile, paying attention to the control and optimization of hospitalization days can help improve the prognosis of patients. In addition, monitoring renal function indicators and careful selection of medication use are also key to reducing the risk of short-term mortality.

This study had some limitations. Firstly, although we have included data from two hospitals, the study period was relatively short and the number of included patients was relatively small, which inevitably leads to some biases (such as Incident Probability bias, sampling error). The existing sample size may lead to the neglect of certain factors that affect the occurrence and prognosis of AAD, such as age, underlying diseases, etc. Due to racial differences, we had to convert continuous variables into binary variables, which, although beneficial for the applicability of nomograms, also led to the occurrence of some biases. Secondly, due to limitations in sample size, we had to exclude indicators with high missing values, potentially resulting in an incomplete assessment of short-term mortality risk using only the five mentioned risk factors. Additionally, the lack of access to patients’ medical records limited our understanding of their treatment process and reduced the reliability of our constructed nomogram. In the future, by increasing the sample size and improving the follow-up mechanism, more meaningful conclusions may be obtained.

## Conclusion

In conclusion, this study developed a prediction model based on gender, length of hospitalization, BUN_max, use of adrenaline, and amiodarone. The nomogram could help to identify high-risk patients who may die within one year after discharge, thereby facilitating clinical practice and providing valuable references for doctors focusing on follow-up and intervention strategies of AAD patients.

## Data Availability

The data underlying this article will be shared on reasonable request to the corresponding author.
